# Using a System-Based Monitoring Paradigm to Assess Fatigue during Submaximal Static Exercise of the Elbow Extensor Muscles

**DOI:** 10.3390/s21041024

**Published:** 2021-02-03

**Authors:** Kaci E. Madden, Dragan Djurdjanovic, Ashish D. Deshpande

**Affiliations:** Department of Mechanical Engineering, The University of Texas at Austin, Austin, TX 78712, USA; kaci.madden@utexas.edu (K.E.M.); dragand@me.utexas.edu (D.D.)

**Keywords:** human fatigue monitoring, neuromuscular fatigue, surface electromyography time-frequency signal analysis, time-series modeling, autoregressive moving average model with exogenous inputs, isometric contraction, elbow extension

## Abstract

Current methods for evaluating fatigue separately assess intramuscular changes in individual muscles from corresponding alterations in movement output. The purpose of this study is to investigate if a system-based monitoring paradigm, which quantifies how the dynamic relationship between the activity from multiple muscles and force changes over time, produces a viable metric for assessing fatigue. Improvements made to the paradigm to facilitate online fatigue assessment are also discussed. Eight participants performed a static elbow extension task until exhaustion, while surface electromyography (sEMG) and force data were recorded. A dynamic time-series model mapped instantaneous features extracted from sEMG signals of multiple synergistic muscles to extension force. A metric, called the Freshness Similarity Index (FSI), was calculated using statistical analysis of modeling errors to reveal time-dependent changes in the dynamic model indicative of performance degradation. The FSI revealed strong, significant within-individual associations with two well-accepted measures of fatigue, maximum voluntary contraction (MVC) force ($r_{rm}=-0.86$) and ratings of perceived exertion (RPE) ($r_{rm}=0.87$), substantiating the viability of a system-based monitoring paradigm for assessing fatigue. These findings provide the first direct and quantitative link between a system-based performance degradation metric and traditional measures of fatigue.

## 1. Introduction

### 1.1. Background

Fatigue, commonly defined as “any exercise-induced reduction in the ability of a muscle to generate force or power” [1], is a complex accumulation of psychological and physiological processes that impair muscle function and diminish the capacity of the central nervous system to activate muscles [1,2,3]. Neuromuscular fatigue presents a major obstacle for achieving desired performance in a variety of circumstances. For healthy individuals in physically demanding professions (e.g., astronauts, soldiers, athletes, etc.), prolonged periods of training and operations are known to adversely affect task efficiency [4], movement accuracy [5], and performance [4], while also increasing susceptibility to overuse injuries [4]. For patients with neurological or cerebrovascular diseases, such as stroke, multiple sclerosis, and Parkinson’s disease, fatigue is also a typical and potentially debilitating symptom [6,7]. Thus, assessing fatigue has important implications for preventing neuromuscular injury [8], optimizing training loads [9], and guiding effective, individualized treatment strategies for rehabilitation [7].

In a clinical setting, standard methods for assessing fatigue rely upon self-reported questionnaires or rating scales [1,10] that capture how an individual experiences fatigue. Mental fatigue can be experienced as an increase in the perceived effort to complete a task [11] or a reduction in motivation and concentration [1]. Ratings of perceived exertion (RPE) [12,13,14] are used to study mental fatigue in healthy and affected populations. A higher perception of effort is known to limit exercise tolerance [13] and adversely affect physical performance during endurance tasks [11,14]. Although subjective rating scales contain valuable information, they are indirect measures of fatigue that provide qualitative information with low-resolution [10]. Moreover, self-perceived fatigue is not always accompanied by a loss of force-producing capacity [6,11,14] or changes in physiological variables [13,14], especially during endurance tasks.

A decline in maximum voluntary contraction (MVC) force has become a “gold standard” [15] indicator for confirming the occurrence of fatigue in the physiological sciences [1,14,15] because it can directly quantify a loss in force-generating capacity. Despite their value as objective assessment tools, MVC force measures are often taken immediately before and after a bout of exercise and only capture the overall mechanical manifestation of fatigue. Consequently, they lack valuable insight regarding the progression of fatigue during the task itself, including the underlying physiological processes that contribute to the degraded performance of the neuromuscular system. Neuromuscular fatigue can be identified by measuring the evoked force from twitch responses after electrically stimulating muscles during maximal or submaximal voluntary contractions [16]. However, this technique is also applied before and after a fatiguing exercise.

Surface electromyography (sEMG) has been widely used to address this issue by enabling the continuous measurement of muscle activity during exercise. Since fatigue begins to accumulate at the start of a muscle contraction and continuously progresses throughout the exercise [17], changes in the sEMG signal can reveal indications of localized muscle fatigue long before a decline in force or power output occurs [1,2,18]. For instance, during sustained contractions at submaximal force levels, a progressive increase in sEMG amplitude and compression of the sEMG signal spectrum can be detected [2,17,18]. Fourier-based spectral features extracted from the sEMG signal, such as the mean or median frequency, are the most widely used indices of localized muscle fatigue and have been employed in numerous applications [2,18,19].

Extensive work has been devoted to developing more advanced spectral estimation and signal processing techniques that can accommodate the non-stationary behavior in sEMG signals [20,21]. The majority of these efforts, which are thoroughly discussed elsewhere [21,22,23,24], were devoted to developing fatigue assessment metrics that reflect the localized manifestations of fatigue within a muscle. Thus, these metrics are often univariate, monitored independently for each muscle, and analyzed separately from associated changes in joint movement. Less attention has been paid to developing multivariate metrics that utilize more information from the sEMG signal, aggregate activity from all contributing muscles, and establish a relationship with kinematic or kinetic movement variables. Such metrics would be beneficial for assessing how the neuromuscular system fatigues as a whole during exercise.

### 1.2. Related Literature

Model-based methods that relate sEMG parameters to movement variables have shown success in producing a single, unified metric for monitoring fatigue, overcoming some of the aforementioned issues. Previous studies have applied linear regressions [23], artificial neural networks [23,25,26], linear projection methods [27], and correlations [28] to map net changes in sEMG parameters to overall reductions in power [23] or force [28]. Although promising, these approaches do not continuously monitor changes in the dynamic relationship between sEMG and movement output over time—a relationship that is significantly altered in the presence of fatigue [29]. They also require (i) *a priori* assumptions about the linearity of fatigue progression [23,25,26], (ii) extensive data sets containing the entire time-course of fatigue to train models [23,25,26,27,28], and (iii) reference contractions to probe for fatigue-induced changes in parameters at the beginning and end of an endurance task [28].

Recent studies have approached human performance monitoring using a system-based monitoring paradigm, which is relatively well-known in the machine monitoring community [30]. The system-based approach monitors how the performance of the human neuromusculoskeletal (NMS) degrades during prolonged exercise by continuously tracking changes in the dynamic relationship between sEMG and movement output over time. Musselman et al. were the first to pursue this direction [31]. The dynamic relationship was described using vectorial autoregressive models with exogenous inputs (vARX), which took instantaneous intensity and frequency features from upper-arm sEMG signals as inputs and related them to joint angular velocities as model outputs. The methodology was tested on data from participants performing a repetitive sawing movement until voluntary exhaustion. Xie and Djurdjanovic [32], Madden et al. [33], and Yang et al. [34] modified this work by instead using autoregressive moving average models with exogenous inputs (ARMAX) with second-order muscle dynamics to describe the NMS system during both constant force and repetitive movement tasks. Two additional sEMG features, namely instantaneous variance and entropy, were incorporated as model inputs with either force [32,33], joint velocity [32], or limb displacement [33] serving as outputs, depending on the task.

The models in all four studies [31,32,33,34] were trained with data from the initial portion of the task before fatigue onset to capture the system dynamics during a normal, unfatigued state. Progressive changes in system behavior were evaluated by tracking the divergence of model prediction error distributions between the unfatigued state and subsequent periods of time. Statistically significant trends in a divergence measure, referred to as either the freshness similarity index (FSI) [32,33], fatigue index [34], or global freshness index [31], provided evidence that performance degradation occurred during the exercises.

The system-based monitoring paradigm overcomes the limitations imposed by alternative, model-based approaches to monitoring fatigue [23,25,26,28]. However, the previous system-based monitoring efforts [31,32,33,34] have not established a formal association between performance degradation and fatigue. Although the studies verified their findings using trends in sEMG features to reveal indications of localized muscle fatigue in individual muscles, the indices in [31,32,33,34] are constructed as global measures of how the performance of the entire NMS system changes over time. Thus, to claim that a system-based monitoring approach is a viable method for monitoring fatigue, further research is needed to formally associate the performance degradation index with well-established fatigue measures that quantify a global reduction in force-producing capacity [1,15] and heightened perception of exertion [12], rather than changes intramuscular mechanisms. Furthermore, modifications can be made to the system-based paradigm used in these previous works [31,32,33,34] to produce sEMG features that are more representative of neural activation signals to the NMS system, provide a complete representation of the NMS system by incorporating all contributing muscles, and facilitate online performance assessment.

To this end, the primary aim of this work is to firmly establish the viability of the system-based monitoring paradigm for assessing fatigue by relating the performance degradation index to well-accepted measures of fatigue that capture changes in force-generating capacity (MVC force) and self-perceived fatigue (RPE). We present a methodology, modified from previous works, to generate a sensitive and concise index of performance degradation (FSI) occurring across multiple muscles and sensor sources during a submaximal static exercise. We then substantiate its viability for assessing fatigue by evaluating within-individual associations between the FSI and measures of MVC force and RPE. We discuss the improvements made to the paradigm to facilitate its use as an online assessment tool and more accurately represent changes occurring in the NMS. The results of this work have promising implications for informing new methods of monitoring fatigue. Tracking fatigue-related changes in performance may lead to more personalized training regimens and therapeutic modalities for rehabilitation. Interventions involving robotic exoskeletons present an especially promising application of the system-based monitoring paradigm because these devices possess high-resolution sensors that can collect physiological, dynamic, and kinematic measures in real-time.

## 2. Materials and Methods

### 2.1. Participants

Eight healthy, right-handed men (26.6 ± 6.1 years, 76.2 ± 12.4 kg, 178.9 ± 6.6 cm) with no known neurological disorders were recruited from the university population to participate in the study. All participants were fully informed of any risks associated with the experiments before giving their informed written consent to participate in the investigation. The study was conducted in accordance with the Declaration of Helsinki [35], and the experimental procedure was approved by the Internal Review Board organized by the Office of Research Support at The University of Texas at Austin under the protocol number 2013-05-0126.

### 2.2. Experimental Setup

**Figure 1:**
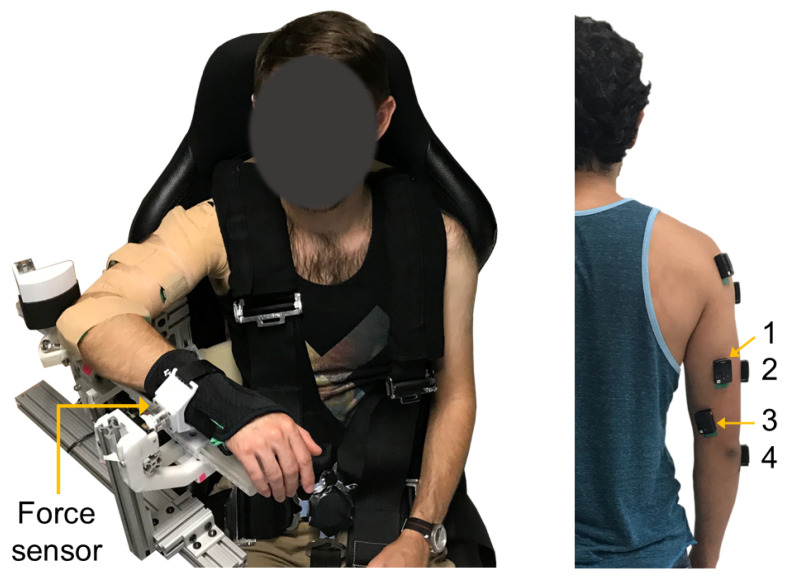
Experimental setup. (**Left**) Exoskeleton testbed. (**Right**) sEMG sensor placement: (1) long, (2) lateral, and (3) medial heads of the triceps brachii, and (4) anconeus muscles.

Participants were seated in a high-back chair with a five-point harness that restrained their waist and shoulders (Figure 1). A single-degree-of-freedom exoskeleton testbed was grounded to the base of the chair and used for testing. The device consists of an upper arm linkage, capstan drive elbow joint, and lower arm linkage with a wrist cuff. The chair and link lengths were adjusted to accommodate each participant. The participants’ upper arm was positioned at 90${}^{\circ }$ of flexion and 45${}^{\circ }$ of horizontal abduction, and their elbow placed in 90${}^{\circ }$ of flexion. The medial epicondyle of the participants’ humerus was aligned with the exoskeleton elbow joint axis, and the forearm was placed in a neutral position. The wrist cuff was positioned below the participants’ ulnar styloid process and securely attached their forearm to the exoskeleton. The lower arm linkage of the exoskeleton was grounded to the chair using a mechanical structure to prevent the elbow joint from rotating during the isometric contractions described in Section 2.3. As a result, the robot actuator remained unpowered during experimentation. A multi-axis force/torque sensor mounted to a linear sliding joint was housed between the wrist cuff and exoskeleton linkage and used to measure the participants’ elbow extension force. The linear slider allowed passive travel in the direction parallel to the ulna bone to minimize off-axis forces due to robot-human misalignment [36].

### 2.3. Experimental Protocol

Experiments were carried out in the ReNeu Robotics Laboratory at the University of Texas at Austin. All participants performed the same experiment on two days separated by 72 h of rest [37,38] in a temperature-controlled room set to 70${}^{\circ }$. Both sessions were performed at the same time of day and followed the same general protocol, which consisted of three elbow extension tasks: (1) baseline maximum voluntary contractions (MVCs) (2) a constant-force endurance task sustained at 30% MVC until exhaustion, and (3) a follow-up MVC. Only results from the first session are reported in this paper. Participants were instructed to refrain from consuming caffeine on the day of testing [39] and exercising 24 h before the experiment.

Before testing, the participants performed isometric elbow extension, elbow flexion, shoulder flexion, shoulder abduction, and shoulder extension contractions for which they were asked to maximally and submaximally exert force. During testing, participants were provided with real-time visual feedback of their elbow extension force, in the form of a gauge display, on a computer monitor placed at eye-level. For the MVCs, participants were instructed to gradually increase extension force output from zero to maximum over a 3 s period and maintain their maximal force for an additional 2–3 s. The participants were given strong verbal encouragement to provide maximal effort during each contraction. At baseline, a minimum of three MVCs separated by one minute of rest were performed. If peak forces from two of the three MVCs were not within 5%, additional trials were performed until this criterion was met. The trial consisting of the highest value was retained and considered the MVC force. Participants then rested for at least eight minutes to minimize residual fatigue from the MVC tasks.

Before the endurance task, each participant was familiarized with their MVC levels by performing brief elbow extension contractions at various force levels (i.e., 30% and 60% MVC). For the endurance task, participants performed a sustained, isometric contraction at 30% MVC until their force fell below 10–15% of the target value [39,40]. In related works examining fatigue, the MVC thresholds of the isometric contractions vary between 25–35% [28,39,40,41]. The contraction level for evaluation was set to 30% MVC for this study, as it is the average between these ranges. The target force (30% MVC) and the participant’s actual extension force were displayed on the computer monitor. Participants matched and tracked the target line for as long as possible and were verbally encouraged to maintain a steady force output. Every 30 s, participants reported a rating of perceived exertion (RPE) using the Borg CR-10 scale [12]. These ratings ranged from 0 (“no exertion at all”) to 10 (“maximal exertion”). Immediately after termination of the endurance task, participants reported a final RPE and performed a follow-up MVC to determine the amount of fatigue induced.

### 2.4. Data Acquisition

A Delsys Trigno Wireless EMG system (Delsys Inc., Boston, MA, USA) was used to collect sEMG activity from the triceps brachii (long, lateral, and medial heads), anconeus, biceps brachii, brachioradialis, and deltoid (anterior, middle, and posterior) muscles. The scope of this paper requires analysis of only the muscles that extend the elbow, that is, the triceps brachii and anconeus (Figure 1). Participants’ body hair was shaved, and skin lightly abraded with a pumice stone then cleansed with isopropyl alcohol to ensure good skin-to-electrode contact before sEMG sensor placement. Electrodes were positioned over each muscle according to European recommendations for Surface Electromyography for Non-Invasive Assessment of Muscles (SENIAM) [42]. Elbow extension forces were measured with a multi-axis force/torque sensor (ATI, Nano25). An xPC Target (Mathworks, MATLAB module) running Simulink Real-Time and hosting NI data acquisition (NI DAQ) boards (National Instruments, Inc., Austin, TX, USA) synchronously recorded all data at 1 kHz.

### 2.5. Data Processing

Raw sEMG signals were bandpass filtered from 10 to 400 Hz [43,44] using a 4th order Butterworth filter (zero-lag, non-causal) [45], then demeaned [46] to remove the DC offset. Data from the force/torque sensor was low-pass filtered using a 4th order Butterworth filter (zero-lag, non-causal) with a 6 Hz cutoff frequency. The processed sEMG and force measures are used in Section 2.6.1 and Section 2.6.2.

### 2.6. System-Based Monitoring

#### 2.6.1. sEMG Feature Extraction

**Figure 2:**
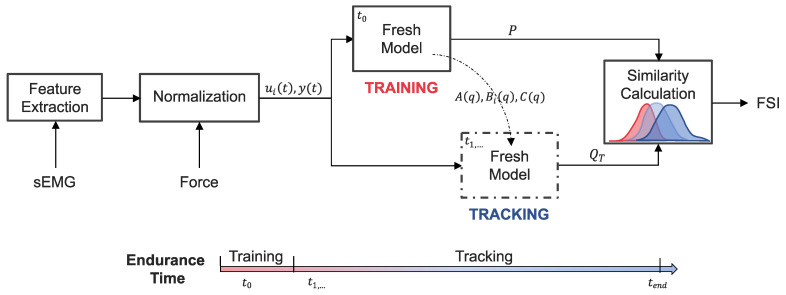
System-based monitoring workflow. Features are extracted from the surface electromyography (sEMG) signals of each muscle. The sEMG features and elbow extension force are then normalized and used as the inputs ($u_{i}\left(t\right)$) and output (y(t)) to a dynamic time-series model. Training data from the start of the endurance task ($t_{0}$) is used to identify the polynomial coefficients ($A\left(q\right),B_{i}\left(q\right),C\left(q\right)$) of the “Fresh Model” and calculate a reference distribution (*P*) of one-step ahead prediction errors. The remaining endurance task data ($t_{1,\ldots ,end}$) is incrementally introduced to the tuned “Fresh Model” for which updated prediction error distributions ($Q_{T}$) are calculated at each time step, *T*. The overlap between *P* and $Q_{T}$ is evaluated to obtain a time-series of freshness similarity index (FSI) values that quantify performance degradation.

The first step in the system-based monitoring workflow (Figure 2) involves extracting features from the filtered sEMG signals [47] that capture how the signal energy changes in both the time and frequency domains. Cohen’s class of time-frequency distributions (TFD) was used to obtain a two-dimensional probability density function, C(t,ω), describing the joint distribution of energy of the sEMG signal, s(t), over time, *t*, and frequency, ω, where

(1) $$\begin{array}{c} C(t,\omega )=\frac{1}{4\pi^{2}}\cdot \\ \overset{+\infty }{\underset{-\infty }{\;\int \int \int \;}}s^{*}\left(u-\frac{1}{2}\tau \right)s\left(u+\frac{1}{2}\tau \right)\phi \left(\theta ,\tau \right)e^{-j(\theta (t-u)+\tau \omega )}d\tau dud\theta , \end{array}$$

with $s^{*}\left(t\right)$ signifying the complex conjugate of s(t) and ϕ(θ,τ) denoting the so-called TFD kernel. The binomial kernel, a signal independent member of the reduced interference distribution family of kernels, was used for this analysis due to its desirable mathematical properties [31].

Calculation of the zero- and first-order moments (i.e., $<f^{0}|t>$ and $<f^{1}|t>$) of C(t,ω) provide the instantaneous energy and instantaneous mean frequency of the sEMG signal, respectively, with

(2) $$<f^{0}|t>=\int_{-\infty }^{+\infty }C\left(t,\omega \right)d\omega =|a_{i}{\left(t\right)|}^{2}$$

(3) $$\begin{array}{c} <f^{1}|t>=\int_{-\infty }^{+\infty }\frac{C(t,\omega )}{<f^{0}|t>}\omega d\omega =f_{im}\left(t\right), \end{array}$$

where $a_{i}\left(t\right)$ is the instantaneous amplitude—a parameter that is approximately equal to the RMS amplitude of the sEMG signal [48,49]. The instantaneous mean frequency, labeled as $f_{im}\left(t\right)$, and instantaneous amplitude, $a_{i}\left(t\right)$, are widely used as myoelectric indicators of fatigue. As a result, significant decreasing trends in $f_{im}\left(t\right)$ and increasing trends in $a_{i}\left(t\right)$ during the constant-force endurance task would substantiate the presence of localized muscle fatigue [2,18,19].

Previous system-based monitoring studies [31,32,33] used the instantaneous energy ($<f^{0}|t>$), rather than $a_{i}\left(t\right)$, as an input to the dynamic model described in Section 2.6.3. However, we adopted $a_{i}\left(t\right)$ because it is analogous to the RMS amplitude of the sEMG signal that reflects changes in “neural drive” due to fatigue [1]. Moreover, the square root calculation in (2) attenuates the high magnitude spikes produced when computing the zero-order moment, which is apparent in [31]. Previous works also extracted two additional sEMG features, representing the second-order moment and entropy of the signal, to be used as model inputs [32,33,34]. When including these features in our dynamic model, the performance degradation metric described in Section 2.6.4 did not significantly change. Therefore, we reduced the complexity of our model by restricting the number of model inputs to include only $a_{i}\left(t\right)$ and $f_{im}\left(t\right)$ for each muscle.

#### 2.6.2. Normalization

**Figure 3:**
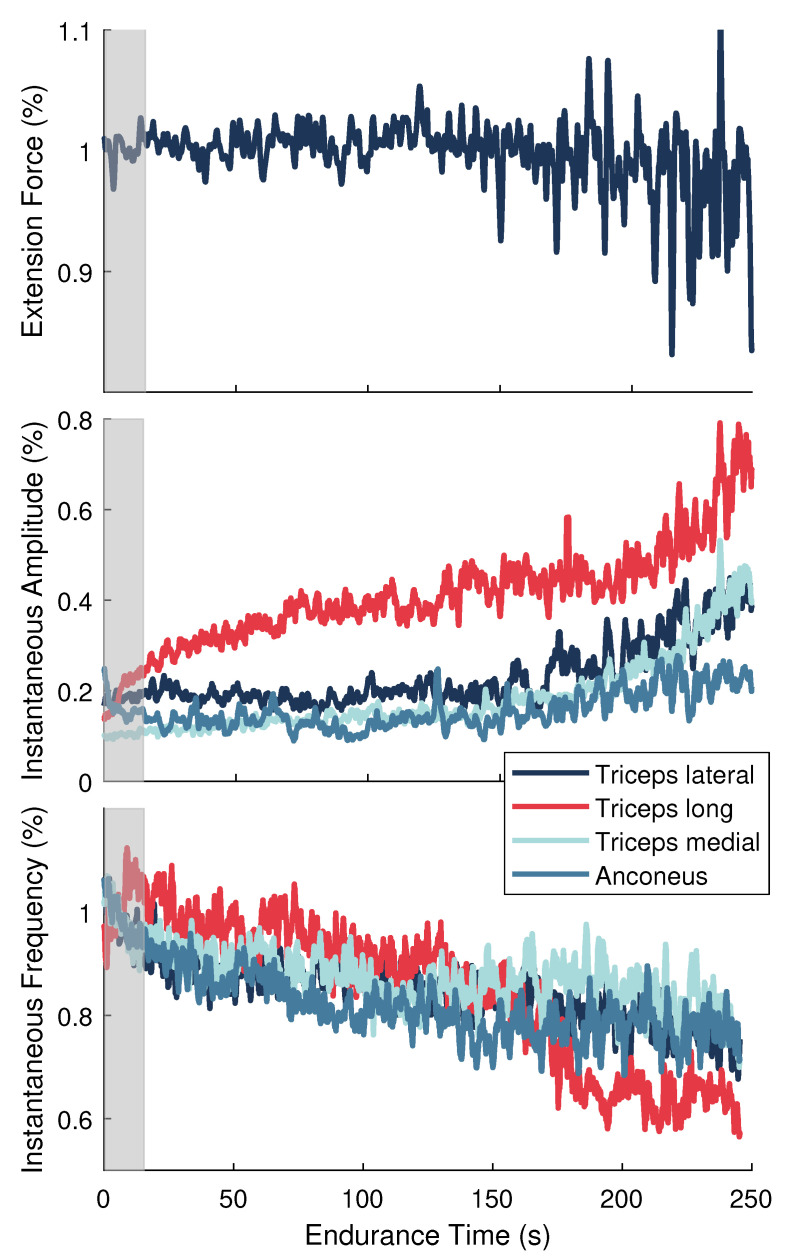
Normalized signals for a single representative participant during the endurance task. (**Top**) Elbow extension force. (**Middle**) Instantaneous amplitude ($a_{i}\left(t\right)$) and (**Bottom**) instantaneous frequency ($f_{im}\left(t\right)$) features for the elbow extensor muscles. Gray shaded area signifies the training data set.

Data from the MVC and endurance tasks were smoothed using 10 ms and 1.5 ms sliding windows, respectively. Maximal values obtained over a 1.5 s period around the peak MVC reference force were determined for each muscle and used to normalize the corresponding $a_{i}\left(t\right)$ signals from the endurance task. Force and $f_{im}\left(t\right)$ signals from the endurance task were normalized to their average values during the initial 10 s of the endurance task. All signals were then downsampled to 100 Hz. This procedure prepared the data to be used in the model described in Section 2.6.3 and shown in Figure 2. Figure 3 depicts the force and sEMG features after normalization for one representative participant.

The normalization strategy presented in this work was another improvement made to previous system-based monitoring attempts, which used data from the entire endurance task to normalize the signals [32,33]. By scaling $a_{i}\left(t\right)$ to MVC values and $f_{im}\left(t\right)$ to initial values, our normalization approach produced signals that are more representative of neural activation signals and the frequency-based sEMG indices found in the literature for assessing localized muscle fatigue. Moreover, our approach could be employed for online performance assessment because the only data needed for normalization was collected at the beginning of the experiment (i.e., baseline MVC contractions performed before testing and the initial few seconds of the endurance task).

#### 2.6.3. Modeling

Human skeletal muscle can be considered a viscoelastic system whose physiological input is a neural signal and output response is a generated force [50]. Thus, the normalized sEMG features extracted from the triceps brachii (long, lateral, and medial heads) and anconeus muscles were used as neural inputs to a dynamic model whose output is elbow extension force. The dynamics were represented using an autoregressive moving average model with exogenous inputs (ARMAX). This form of parametric system identification approximates force as a linear transformation of sEMG features and noise terms and can be expressed as

(4) $$A\left(q\right)y\left(t\right)=\sum_{i=1}^{n_{u}}B_{i}\left(q\right)u_{i}\left(t\right)+C\left(q\right)e\left(t\right),$$

where the system output, y(t), is the elbow extension force, the system input, $u_{i}\left(t\right)$, is an $n_{u}$× 1 vector of the normalized sEMG features, and e(k) is the model disturbance considered to be zero mean Gaussian process noise. Since two sEMG features ($a_{i}\left(k\right)$ and $f_{im}\left(k\right)$) were extracted from each muscle, $n_{u}=8$. The polynomials *A*, $B_{i}$, and *C* are expressed in terms of the time-shift operator, $q^{-1}$, and can be written as

(5) $$\begin{array}{c} A(q)=1+a_{1}q^{-1}+\ldots +a_{n_{a}}q^{-n_{a}}\\ B_{i}\left(q\right)=b_{1}+b_{2}q^{-1}+\ldots +b_{n_{b}}q^{-n_{b}+1}\\ C(q)=1+c_{1}q^{-1}+\ldots +c_{n_{c}}q^{-n_{c}}, \end{array}$$

where $n_{a}$, $n_{b}$, and $n_{c}$ are their respective orders. The model was structured such that each muscle is considered a second-order dynamic system [32]. This approach is in line with Gottlieb and Agarwal [50] and Thelen et al. [51] who found that a second-order system can adequately describe the functional relationship between sEMG and force [50] or joint torque [51]. Thus, the orders of the polynomials were selected to be 8 for A(q) and $B_{i}\left(q\right)$ and 7 for C(q). Separate models were trained for each user with data selected from the initial 15 s of the endurance task (Figure 2 and Figure 3). This training data set captures the state of the users before significant fatigue could develop. Thus, the trained model, referred to as the “fresh model” (Figure 3), captures the system dynamics corresponding to the user’s least degraded, or least fatigued, state.

#### 2.6.4. Performance Tracking

Using the training data set, a reference distribution, *P*, of 1-step ahead prediction errors was generated by the “fresh model” (Figure 2). The remaining data from the endurance task was segmented into *T* epochs that were 4 s in length. The endurance time for each participant determined the total number of epochs. These data segments were sequentially presented to the “fresh model” to calculate the latest 1-step ahead prediction error distributions, $Q_{T}$. The Fidelity similarity metric [52,53] was then calculated to evaluate the amount of overlap between the reference and updated distributions over time. The metric, which is referred to as the Freshness Similarity Index (FSI), is defined as

(6) $$\begin{array}{c} FSI=1-\sum_{i=1}^{N}\sqrt{P\left(i\right)Q_{T}\left(i\right)} \end{array}$$

and ranges from 0 to 1, where values near 0 indicate a high degree of similarity and those close to 1 suggest little similarity. For context, if the dynamic system remains unaltered with time, the updated distributions will be comparable to the fresh distribution. However, if the system dynamics change due to fatigue or injury, for example, the updated distribution will shift or change shape, reducing the amount of overlap with the fresh distribution. Thus, the FSI is a metric that reflects how the ARMAX approximation of the system dynamics degrades over time with respect to a normal, unfatigued state.

Previous system-based monitoring studies used similarity/divergence measures, including Matusita’s overlap coefficient measure [31,32,33] and the Kullback-Leibler divergence measure [33]. However, the Fidelity similarity metric was used in this work due to its superior sensitivity to changes in modeling errors for the data in this study. All data processing and modeling was conducted using MATLAB software (R2017b) [54].

### 2.7. Statistical Analysis

A paired samples t-test was used to test for differences between baseline (pre-endurance task) and follow-up (post-endurance task) MVC forces, and Cohen’s d was used to calculate the effect size between time points. A one-factor repeated measures analysis of variance (RM-ANOVA) was used to evaluate mean differences in RPE scores collected after the first, middle, and last 30 s of the endurance task. For each sEMG feature, a two-factor RM-ANOVA was used to test for differences across time and within muscles using average values over the first, middle, and last 30 s of the endurance task. FSI was quantified in two ways. For statistical analysis, averages over the first, middle, and last 30 s of the endurance task were used in a one-factor RM-ANOVA to evaluate mean differences over time. For graphical representation, average FSI values over each 1% of the endurance time were presented. A Greenhouse-Geisser correction was applied to correct for violations of sphericity when Mauchly’s test was significant. Significant main effects were further examined using estimated marginal means with a Tukey-Kramer adjustment for multiple comparisons.

Within-subject correlations [55] were performed using repeated-measures correlation (rmcorr) [56] analysis to evaluate the associations between FSI and measures of force-generating capacity (MVC force) and self-perceived fatigue (RPE scores). Although associations between parameters may typically be analyzed using simple correlations that quantify between-subject associations, within-subject associations are more important to this study because FSI is an individual-specific metric. Rmcorr analysis also provides benefits over simple correlation techniques when considering the change in variables over time. Multiple data points per participant can be used in a rmcorr, whereas simple correlations require time-series data to be aggregated so that all observations are independent of each other. As a result, rmcorr can yield much greater power than simple correlation methods and detect relationships between variables that might otherwise be masked when using aggregated data. Two rmcorr analyses were used to estimate linear models with subject-specific intercepts relating FSI to MVC force and FSI to RPE scores. Paired data from the start (i.e., pre-endurance task/first 30 s) and end (i.e., post-endurance task/last 30 s) of the endurance task was used for the rmcorr between MVC force and FSI. Paired data from the first, middle, and last 30 s of the task was used for the rmcorr between RPE and FSI. The resulting rmcorr coefficient ($r_{rm}$) quantified the common within-individual association between variables.

Although the results from the rmcorr analyses were used to evaluate the FSI metric, between-subject associations were also reported based on simple correlations. Pearson’s product-moment correlation coefficient (*r*) was used to assess the association between FSI and MVC force. The Spearman rank correlation coefficient ($r_{S}$) was used to evaluate the relationship between FSI and RPE because the RPE scores were treated as ordinal data. To minimize biases introduced by the time-dependency among data points, the paired data was aggregated into difference scores representing the overall change in measures from the start (i.e., pre-endurance task/first 30 s) to the end (i.e., post-endurance task/last 30 s) of the endurance task. Shapiro-Wilk tests verified that all difference scores were normally distributed. We hypothesized that FSI would be negatively correlated with MVC force and positively correlated with RPE.

Using the guidelines presented in [57], correlation coefficients were interpreted as very strong (r ≥ 0.9), strong (0.7 ≤ r < 0.9), moderate (0.5 ≤ r < 0.7), weak (0.3 ≤ r < 0.5), and negligible (r < 0.3). All statistical analyses were conducted using R software (3.6.1) [58]. RM-ANOVAs and follow-up tests were analyzed using the *afex* and *emmeans* packages. Within-subject correlations were determined using the rmcorr package [56]. Statistical significance was set at p<0.05 for all testing. Data are reported as mean ± standard error of the mean (SE) unless stated otherwise.

## 3. Results

### 3.1. Confirmation of Fatigue

**Figure 4:**
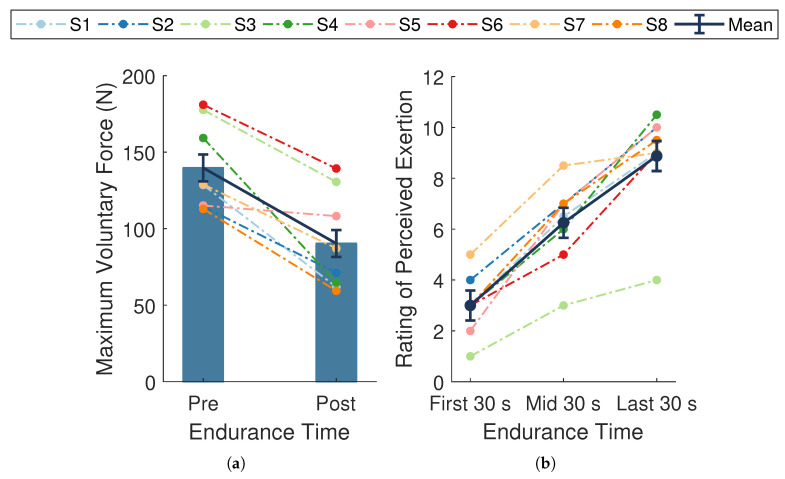
Measures of fatigue. (**a**) Maximal voluntary contraction (MVC) forces taken at baseline (pre-endurance task) and follow-up (post-endurance task). (**b**) Ratings of perceived exertion (RPE) during the first, middle, and last 30 s of the endurance task. Dark blue bars and data points connected by solid lines are means ± SE. Dotted lines represent data from a single participant (n=8) whose assigned color is consistent across figures. MVC force significantly declined (p<0.001, d=−1.99) and RPE significantly increased over time (p<0.001, $\eta_{p}^{2}=0.91$).

The average endurance time across participants was 287.4 ± 28.0 s. The average MVC force at baseline was 139.8 ± 10.1 N and significantly declined by 49.5±8.8 N, or 35.6±6.1%, (t(7)=−5.63, p<0.001,d=−1.99; Figure 4a) at follow-up. This substantial decline in MVC force from baseline to follow-up verifies that the experimental protocol successfully induced fatigue across participants.

A significant change in mean RPE scores occurred during the endurance task (F(2,14)=74.15, p<0.001, $\eta_{p}^{2}=0.91$; Figure 4b). Post hoc pairwise comparisons revealed significant differences between all measured time points (all *p*-values <0.001). There was an overall mean increase of 5.9±0.5 across participants, with slightly higher changes in scores during the first half (3.2±0.5) compared to the last half (2.6±0.5) of the task. The overall rise in RPE scores indicates the endurance task became increasingly more difficult for the participants as time progressed, providing evidence of self-perceived fatigue.

### 3.2. Evidence of Localized Muscle Fatigue

**Figure 5:**
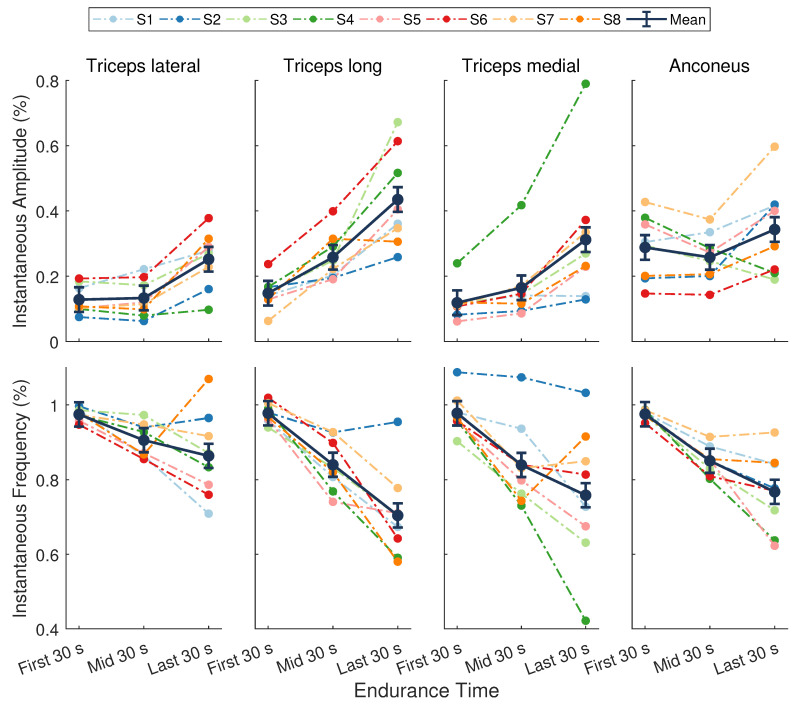
Normalized sEMG features from the elbow extensor muscles. (**Top**) Instantaneous amplitude ($a_{i}\left(t\right)$) and (**Bottom**) instantaneous mean frequency ($f_{im}\left(t\right)$). Dark points separated by solid lines are means ± SE for the first, middle, and last 30 s of the task. Dotted lines represent data from a single participant (n=8) whose assigned color is consistent across figures. There was a significant main effect of time for $f_{im}\left(t\right)$ (p<0.001, $\eta_{p}^{2}=0.83$) and $a_{i}\left(t\right)$ (p<0.001, $\eta_{p}^{2}=0.83$).

A significant main effect of time was found for the instantaneous amplitude ($a_{i}\left(t\right)$) during the endurance task ($F\left(1.38,9.68\right)=116.65,p<0.001,\eta_{p}^{2}=0.83$; Figure 5). No significant differences were present across muscles (F(2.04,14.27)=3.48,p=0.058, $\eta_{p}^{2}=0.33$), nor was there a muscle by time interaction (F(1.94,13.58)=3.26,p=0.071, $\eta_{p}^{2}=0.32$). The mean $a_{i}\left(t\right)$ across all muscles at the beginning, midpoint, and end of the task was 0.17 ± 0.02, 0.2 ± 0.02, and 0.34 ± 0.02, respectively. There was an average increase of 16 ± 1% (p<0.001) over the course of the task, with a greater increase in $a_{i}\left(t\right)$ during the second half of the task (13 ± 1%, p<0.05) compared to the first half (3 ± 1%, p<0.001).

There was a significant main effect of time for the instantaneous mean frequency ($f_{im}\left(t\right)$) during the endurance task ($F\left(1.19,8.34\right)=33.97,p<0.001,\eta_{p}^{2}=0.83$; Figure 5). There were no significant differences across muscles ($F\left(1.88,13.14\right)=2.82,p=0.098,\eta_{p}^{2}=0.29$), nor was there a muscle by time interaction ($F\left(2.80,19.57\right)=2.55,p=0.089,\eta_{p}^{2}=0.27$). The mean $f_{im}\left(t\right)$ across all muscles during the first, middle, and last 30 s was 0.98 ± 0.02, 0.86 ± 0.02, and 0.77 ± 0.02, respectively. On average, the decrease in $f_{im}\left(t\right)$ during the first half of the task (12 ± 2%, p<0.001) was slightly greater than the decrease during the second half of the task (9 ± 2%, p<0.05), resulting in an overall decline from start to end of 20 ± 2% (p<0.001).

The average increase in $a_{i}\left(t\right)$ coupled with a decrease in $f_{im}\left(t\right)$ across muscles indicates that significant localized fatigue developed in the elbow extensor muscles during the endurance task. These trends in sEMG features can be attributed to central and peripheral nervous system mechanisms and intramuscular adaptations [2,17,18]. Our results are consistent with other studies that evaluated the elbow extensor muscles in male participants during sustained isometric contractions [39,40]. For an isometric endurance task held at 25% MVC, Krogh-Lund and Jorgensen [40] found that the median frequency decreased almost linearly in the medial head of the triceps brachii. The RMS amplitude also increased in this muscle, showing greater changes in the last half of the contraction compared to the first. These results parallel the average trends across individuals in our study for $f_{im}\left(t\right)$ and $a_{i}\left(t\right)$, respectively, of the triceps medial head (Figure 5, third column). Davidson and Rice [39] observed significant increases in the RMS amplitude of all three triceps heads (medial, lateral, and long) during an isometric endurance task at 20% MVC. The amplitude of the anconeus muscle, however, revealed smaller increases from the start to the end of the task. Moreover, the long head of the triceps displayed the greatest increase in amplitude across participants at the end of the contraction compared to the other muscles when the participants’ shoulder was in 90${}^{\circ }$ of flexion [39]. The average trends in $a_{i}\left(t\right)$ in our study are in agreement with these findings (Figure 5, top row).

The anconeus and long, lateral, and medial heads of the triceps brachii are considered a synergistic muscle group because they all act to extend the elbow [59]. Evidence suggests that these muscles follow a general hierarchic recruitment pattern to preserve energy [60], where the order of activation depends upon the muscle’s size [60], joint articulation [60,61], fiber composition [59,62,63], and level of effort required by the task [60,64]. Following these principles, the anconeus muscle will activate first at low levels of force, followed by the medial head of the triceps brachii. When effort reaches a moderate-to-high level, the lateral head will be recruited next, followed by the long head [60]. When averaged across individuals, the results from our study closely mirror this recruitment strategy (Figure 5, top row). The anconeus displayed the greatest average $a_{i}\left(t\right)$ of all the synergists at the start of the task. During the first half of the task, sEMG of the medial head showed a moderate increase in $a_{i}\left(t\right)$ and the largest decrease in $f_{im}\left(t\right)$. The $a_{i}\left(t\right)$ of the lateral head remained nearly unchanged, while the $f_{im}\left(t\right)$ showed a modest decrease during this period, indicating it may not have been fully recruited yet. During the second half of the endurance task, all muscles showed a steady increase in $a_{i}\left(t\right)$ and decrease in $f_{im}\left(t\right)$, with the long and lateral heads of the triceps brachii showing the greatest mean changes. These results show that the endurance task, whose target force was only 30% MVC, started as a low effort task but progressed to a moderate-to-high effort task that required increased recruitment of all muscles. The average rise RPE confirmed that subjects felt the level of effort required to maintain force increased during the task.

Although a hierarchic recruitment pattern [60] is evident when averaged across participants, considerable inter-individual variation in this strategy was present in our study. For example, some participants (S6) showed the largest changes in sEMG activity for the long head of the triceps, whereas others (S4) revealed more dynamic trends in the medial head (Figure 5). Moreover, trends in the sEMG amplitude of the anconeus muscle varied widely across individuals. Inter-muscular variability was also evident in our study. The fatigue response within a muscle is known to be variable over time [28,65] and often exhibits curvilinear behavior depending on the intensity of the muscle contraction [66] and activation of other synergist muscles. This type of behavior is most notable in the non-linear trends in the instantaneous amplitude of the anconeus muscle and the reversed trends in the triceps brachii heads over the last half of the endurance task for participant S8 (Figure 5).

### 3.3. Trends in Performance Degradation

**Figure 6:**
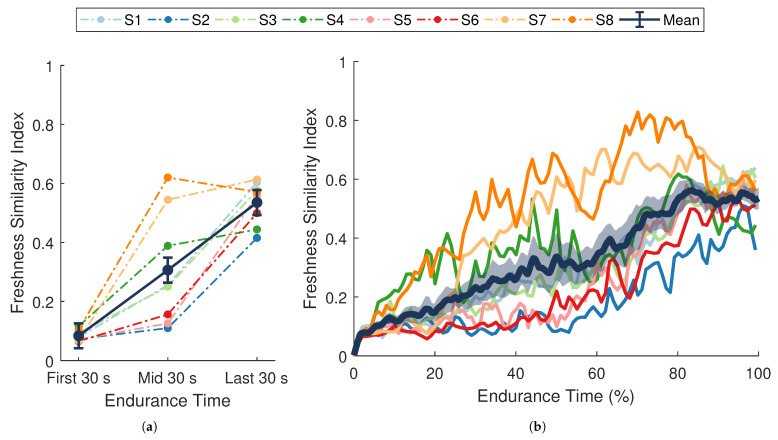
Freshness similarity index (FSI). (**a**) Dark blue data points separated by solid lines are means ± SE for the first, middle, and last 30 s of the task. (**b**) The dark blue line with shaded envelope represents the mean ± SE over each 1% of endurance time. Additional colored lines (dotted in (**a**), solid in (**b**)) represent data from a single participant (n=8) whose assigned color is consistent across figures. FSI increased significantly over time (p<0.001, $\eta_{p}^{2}=0.83$).

There was a significant change in average FSI over the course of the endurance task (F(2,14)=34.17, p<0.001, $\eta_{p}^{2}=0.83$; Figure 6a). Post hoc pairwise comparisons showed significant differences between all time points (all *p*-values <0.001). From the first 30 s to the last 30 s of the task, FSI increased by an average of 0.45±0.05. These results demonstrate that the FSI metric was sensitive to fatigue-induced changes in performance over time. The significant increase observed in the FSI metric (Figure 6) indicates that a progressive temporal change occurred in the dynamic relationship between muscle activity and force output during the endurance task. This general trend coincides with changes in force-generating capacity (MVC force), self-perceived exertion (RPE), and localized muscle fatigue ($f_{im}\left(t\right)$ and $a_{i}\left(t\right)$), suggesting that the phenomenon captured by the FSI metric reflects a degradation in performance over time.

The full time-series of FSI values for each participant are shown in Figure 6b. Although the average trend in FSI is close to linear when averaged across individuals, most participants displayed a non-linear degradation in performance. Moreover, inter-individual differences in the non-linear trends were also apparent. Performance degraded quickly for some participants during the first half of the experiment (S7, S8), whereas others (S2, S5, S6) showed higher rates of change during the latter half.

### 3.4. Relationship Between Measures of Performance Degradation and Fatigue

**Figure 7:**
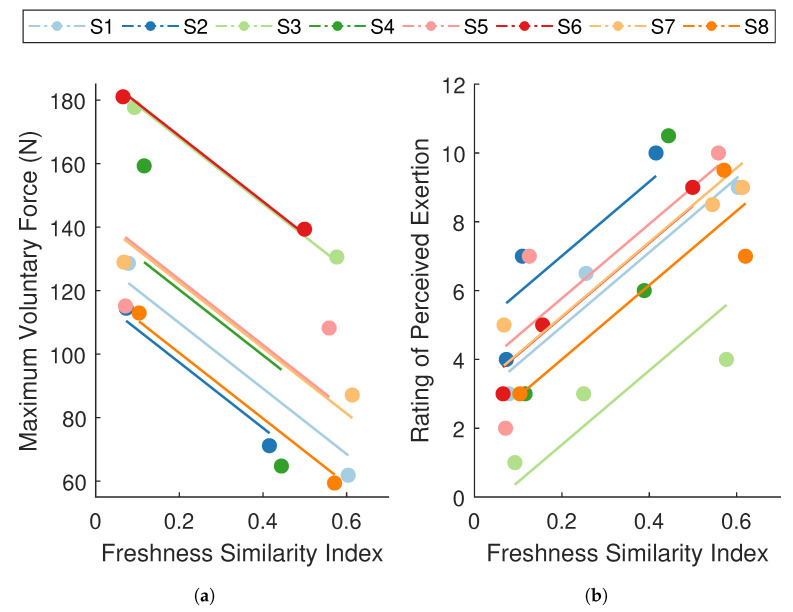
Repeated measures correlations between the freshness similarity index (FSI) and (**a**) maximum voluntary contraction (MVC) force and (**b**) ratings of perceived exertion (RPE). Data points are grouped by participant (n=8), where each color summarizes all observations from one participant and corresponding lines represent the rmcorr fit for that participant. Participant color assignments are consistent with those in other figures. FSI revealed a strong, negative relationship with MVC force ($r_{rm}=-0.86,p<0.01$) and a strong, positive relationship with RPE ($r_{rm}=0.87,p<0.001$).

The rmcorr analyses revealed a strong, negative association between FSI and MVC force ($r_{rm}\left(7\right)=-0.86,95\%$ CI [−0.98,−0.32],p<0.01; Figure 7a), and a strong, positive association between FSI and RPE ($r_{rm}\left(15\right)=0.87,95\%$ CI [0.64,0.96],p<0.001; Figure 7b). These analyses were used to evaluate whether changes in performance degradation were paralleled by changes in mechanical and self-perceived fatigue within the individual. In other words, for a given individual, was an increase in FSI associated with a decrease in MVC force and an increase in RPE. The results indicate that participants who displayed significant performance degradation also experienced a considerable reduction in force-generating capacity and a rise in perceived effort. These strong within-subject relationships between FSI and both well-established measures of fatigue suggest that the degradation in performance captured by the FSI metric is representative of fatigue, thereby substantiating the use of an ARMAX-based monitoring paradigm for assessing fatigue.

Simple correlations between overall changes in FSI and MVC force (r(6)=0.41, p=0.846) and overall changes in FSI and RPE across participants ($r_{s}=-0.34$, p=0.796) were not significant. However, we did not expect to observe between-subject associations. Between-subject associations would suggest that participants with high values of FSI also tend to have high values of RPE and low values of MVC force. However, since the FSI is an individual-specific metric, its absolute value may not be comparable across participants.

## 4. Discussion

### 4.1. Viability of a System-Based Monitoring Approach for Assessing Fatigue

The primary purpose of this study was to substantiate the viability of the system-based monitoring paradigm for assessing fatigue by relating the FSI metric to well-accepted measures of fatigue that capture a net reduction in force-generating capacity (MVC force) and self-perceived fatigue (RPE). The strong within-individual associations between FSI and these traditional measures indicate that the system-based monitoring approach captured fatigue-induced changes in performance, substantiating its use for assessing fatigue. These findings provide the first direct, quantitative link between a system-based approach to monitoring performance degradation and well-accepted measures of fatigue.

To that end, we verified that participants developed fatigue during the endurance task by observing significant reductions in MVC force and increases in RPE. Previous studies that implemented a system-based monitoring paradigm [31,32,33] verified their findings by identifying fatigue in individual muscles using trends in sEMG features. However, trends in the relevant sEMG features reflect localized intramuscular adaptations rather than a global reduction in force-generating capacity [15] or heightened perception of exertion [11,12], whereas the FSI metric is a global representation of system-based performance degradation. Furthermore, in these works, the sEMG features were used as inputs to the vARX and ARMAX models, so comparisons of the sEMG features to the results of the FSI metric might be biased. For these reasons, the present study sought to confirm fatigue using well-accepted global measures of fatigue that are external to the modeling paradigm (i.e., MVC force and RPE) in addition to trends in localized muscle signals. Significant changes in MVC force, RPE, and the sEMG features ($f_{im}\left(t\right)$ and $a_{i}\left(t\right)$) indicate that the participants fatigued during the endurance task.

### 4.2. Improvements to the System-Based Monitoring Paradigm

Additional novelty to the research presented in this paper is in the improvements made to the system-based monitoring paradigm presented in previous works. The modifications, which were specified throughout Section 2.6 and are discussed in more detail below, serve to more accurately represent changes occurring in the NMS and facilitate the use of the system-based monitoring paradigm as an online assessment tool.

We selected the sEMG instantaneous amplitude ($a_{i}\left(t\right)$) as an input to the ARMAX model to minimize the influence of high magnitude transients associated with the instantaneous energy feature used in other studies [31,32,33,34] and provide a comparable sEMG feature to the commonly used RMS amplitude. As such, $a_{i}\left(t\right)$ served to attenuate signal artifacts and better reflected the neural activation of the muscle [1]. To simplify our model structure, we excluded two additional sEMG features from the ARMAX formulation that were used as model inputs in [32,33,34]. These extra features, which capture the variance and entropy of the sEMG signal, provided redundant information and added complexity to our model without improving the sensitivity of the FSI metric to fatigue-related changes in the dynamic relationship between the sEMG features and force.

We normalized the model inputs and outputs in a way that is both consistent with how sEMG signals are processed in the literature [18,67,68] and more suitable for online fatigue assessment compared to previous works [31,32,33]. As a result, the magnitude of the sEMG features fell within predictable bounds, and data from only the baseline MVC contractions and the initial few seconds of the endurance task were needed for scaling. Our strategy would allow for an ARMAX model to be trained using data from short contractions performed before the endurance task, then employed for online monitoring during the endurance task itself. This offers an improvement to previous works whose normalization methods produced model input values that far exceeded the predictable bounds of 0 to 1 [31] or required data from the entire endurance task to obtain the scaling factors [32,33], which would restrict the use of the methodology to post hoc analysis.

Lastly, sEMG features from all elbow extensor muscles were incorporated as inputs to the dynamic model, providing a complete representation of the neuromuscular system responsible for elbow extension. This comprehensive approach extends the capability of previous works, which used a single synergistic calf [32] or forearm [34] muscle to represent the neuromuscular system responsible for isometric plantar flexion and hand grasping, respectively. Although evidence suggests that elbow extensor muscles follow a general hierarchic recruitment pattern, these patterns can vary considerably between individuals and muscles [60], and did vary in our study. Despite these differences, some researchers choose to monitor only one head of the triceps brachii by assuming the sEMG activity from one muscle is representative of the entire synergistic group (i.e., the “equivalent muscle” concept [59]). Although this may be true for brief static contractions [59], the concept does not apply during submaximal contractions held until failure [39]. As a result, assessment approaches that only monitor how one muscle from a synergist group fatigues could underestimate the fatiguing process as a whole. The inclusion of all contributing muscles in our model accommodates the inter-individual differences in muscle recruitment strategies without loss of information by excluding any one particular muscle. Moreover, our approach eliminates the need for *a priori* information regarding muscle fatigability. This is important because the factors contributing to the inter-individual variation (i.e., differences in muscle composition, anatomy, and fitness level) are difficult to measure, making it infeasible to know which muscles will be most fatiguable for a given participant before an experiment is performed.

### 4.3. Performance of the FSI Metric

The FSI metric showed sensitivity to the performance degradation occurring across multiple muscles and sensor sources during an isometric endurance task. The significant increase in FSI demonstrates that the metric was sensitive to changes in the dynamic relationship between sEMG features from the elbow extensor muscles and force that occurred over time. Alterations in this relationship between sEMG amplitude and force are known to occur in the presence of fatigue during isometric tasks [29]. Moreover, by utilizing both amplitude and frequency based sEMG features from each muscle [5], our multivariate ARMAX model effectively detected fatigue-induced changes in the muscle signals [41] and accounted for changes in muscle behavior due to fatigue and those due to altered force production [5].

As a single metric, the FSI also proved to be a concise representation of performance degradation occurring across multiple muscles and sensor sources. Typically, researchers will evaluate fatigue by using sEMG to separately assess intramuscular changes in individual muscles from corresponding alterations in force or movement output. Instead, our system-based methodology uses an ARMAX formulation to represent the neuromusculoskeletal system as an input-output dynamic model and monitors the model’s residuals error over time via the FSI metric. This approach reduces the number of potential monitoring parameters from nine (eight sEMG features and one force signal) to one (FSI), thereby providing a concise representation of fatigue-related degradation in performance.

Most importantly, monitoring the FSI metric also allows for the continuous assessment of fatigue during a task. This can elucidate non-linear performance changes or adaptations that arise over time due to fatigue, as evidenced by the curvilinear evolution of the FSI metric for the majority of individuals in our study. As a result, the system-based monitoring paradigm has clear benefits over MVC-based approaches that must be performed before and after bouts of exercise.

### 4.4. Advantages of a System-Based Monitoring Approach over Alternative Model-Based Techniques for Fatigue Monitoring

The system-based modeling paradigm presented in this paper offers decided advantages over existing model-based fatigue monitoring strategies. First, the methodology does not restrict how performance degradation can evolve over time, thereby allowing for a non-linear progression of FSI. Compared to other model-based fatigue assessment approaches, which utilize *a priori* assumptions that fatigue will progress linearly over time [23,25,26], the methodology is less restrictive and can allow for a more accurate evolution of fatigue-induced changes in performance. Secondly, the ARMAX model used in this study need only be trained on a small data set from the initial portion of the task before fatigue onset. Alternative fatigue modeling attempts require extensive data sets containing the entire time-course of fatigue to train the models [23,25,26,27,28]. This constraint limits the practicality of these approaches due to time-consuming data collection and computationally expensive procedures. The system-based methodology also allows changes in performance to be continually tracked during the endurance task itself, in contrast with other models that use reference contractions to probe for fatigue-induced changes in parameters at discrete time points (e.g., the beginning and end of a task) [28]. Furthermore, our paradigm produces a single overall measure of fatigue, providing an advantage over a model-based technique that used multiple model kernels to evaluate fatigue in each muscle individually [69]. Lastly, our black-box modeling approach requires very few biomechanical assumptions and is capable of performing in a real-time capacity. This offers decided advantages over musculoskeletal modeling approaches that demand knowledge of anatomical parameters and involve time-consuming optimization procedures [70].

### 4.5. Limitations of the Study

Since the system-based modeling paradigm is in a nascent state, the meaning of the absolute value of the FSI is not yet well understood. This is a common issue shared among fatigue metrics [25,27,28,71], however, because the relative change in the parameter over time is generally of more interest than the absolute value of the parameter. The lack of between-subject associations between FSI and other measures of fatigue found in our study verified that the relative change in FSI is not reflecting the differences within individuals. However, with further investigation and participant-specific considerations, FSI values may become more interpretable.

The sample size may be a limitation of the simple Pearson and Spearman correlations used in this work. With a larger group of participants, it may be possible to observe significant between-subject associations between the FSI and both MVC force and RPE. In fact, a multimuscle fatigue score (MMFS) developed in [28] showed weak (r = 0.31) and moderate (r =−0.56) relationships with ratings of perceived fatigue (RPF) and changes in MVC force, respectively, using Pearson product-moment correlations on data from 20 participants. In our study, the sample size was sufficient to evaluate the sensitivity of the FSI to fatigue-related changes in performance using RM-ANOVAs and demonstrate the within-subject associations between FSI and both MVC force and RPE using *rmcorr* analyses. The *rmcorr* analysis can accommodate smaller sample sizes because it uses multiple data points per participant and accounts for non-independence of error between observations using analysis of covariance to statistically adjust for the inter-individual variability [56]. As a result, the degrees of freedom and power will generally be higher using *rmcorr* compared to simple correlations, which use aggregated measures to meet the assumption that data is Independent and Identically Distributed (IID) [56].

This study tested only male participants. However, it is not uncommon for fatigue studies to include only one gender in the participant group [25,28,39,40,65,72]. A related study that evaluated elbow extensor fatigability during a sustained isometric task at 15% MVC until failure reported no differences in endurance time or sEMG amplitude across men and women [73], contrary to observations from other muscle groups that exhibit sex differences [73,74]. Thus, despite the single-gender participant pool used in our study, the findings in [73] provide evidence that our system-based paradigm could account for gender in this muscle group. However, further investigation is necessary to confirm the accuracy of the proposed system-based monitoring paradigm for gender and other factors, such as age.

The ARMAX models were trained on data that was individual- and task-specific, meaning the model parameters, which were estimated for each participant individually during a specific submaximal isometric task, may not be generalizable to other participants or exercises. However, this warrants further investigation. Although model specificity is a shared limitation among other model-based fatigue assessment strategies [23,25,28], personalized models are still essential for making patient-specific clinical decisions [75] or when accurate fatigue monitoring is required, that is, during recovery after musculoskeletal injuries or rehabilitation for patients with neuromuscular disorders [7].

Lastly, insight concerning the specific muscles experiencing fatigue is not reflected in the FSI, as was the case in the model-based approach by [28]. However, the purpose of the system-based monitoring paradigm is to provide a concise measure of fatigue-related changes in performance across multiple muscles and sensor sources. Thus, condensing the number of monitoring parameters down to a single metric allows for a uniform approach to assessing how the entire NMS system responsible for the fatiguing task behaves across individuals. Although only four muscles were considered in the NMS system responsible for elbow extension in this study, the system-based monitoring paradigm is flexible to accommodate any number of inputs.

### 4.6. Applications of the Study

There are many practical applications of this research. The ability to characterize and track fatigue-related changes in neuromuscular system performance during exercise has the potential to inform therapeutic modalities for rehabilitation. It also can become useful when personalizing exercise regimens to target strength or endurance deficits, or by indicating when to stop exercising before significant fatigue leads to the onset of injury. More specifically, this work has the potential to improve fatigue monitoring techniques during robot-aided movement training, which typically apply traditional signal processing methods to analyze localized fatigue of individual muscles using sEMG [10]. Robotic exoskeletons are equipped with high-resolution sensors, such as force sensors and encoders, that can capture kinematic and kinetic measurements reflecting the quality of a user’s movement [76]. In combination with physiological measures, such as sEMG, a system-based monitoring paradigm could fuse the data from these sensor sources and produce a single metric to assess fatigue, such as the FSI. This metric could then be used as an input to an exoskeleton controller that alters the level of robot-applied assistance or resistance to accommodate a patient’s capability and needs [77].

### 4.7. Future Work

Several aspects of the presented methodology are ripe for further exploration to enhance its utility as a diagnostic and monitoring tool. In this work, we chose to use an isometric task to validate that the FSI captures fatigue because it is a simple contraction that does not require the muscle to change length, thereby minimizing the non-stationary behavior of the sEMG signals. Further validation using concentric and eccentric exercises will open the possibility of fatigue monitoring during dynamic movements, which are integral to various therapeutic modalities. Additionally, a formal exploration of how the FSI metric behaves across multiple days of testing and in response to periods of rest and recovery would help prove its effectiveness as a clinical tool. Further advancements to the dynamic model might also lead to improved modeling accuracy and fatigue tracking, especially when expanding the application of this work to more dynamic movements involving multiple joints. In this work, we assumed a linear dynamic relationship between muscle activity and movement output for analytical tractability. Future work could examine the appropriateness of the linear assumption by comparing its accuracy to non-linear dynamic models [78]. In the long run, the approach presented in this paper could be adapted to monitor fatigue in real-time and used to update control laws of robots, e.g., exoskeletons, for optimal human-robot performance.

## 5. Conclusions

This paper presented and validated a framework for continuously assessing fatigue using a system-based monitoring paradigm. The paradigm modeled the dynamic relationship between sEMG features extracted from multiple synergistic muscles to force output, then employed statistical analysis of modeling errors to reveal how performance degraded in each participant over time. The index of performance degradation (FSI) revealed strong, within-individual associations with two well-established fatigue measures, substantiating its applicability as a fatigue monitoring tool. The FSI provided a sensitive and concise representation of the temporal changes in the dynamic relationship between limb force and sEMG parameters during submaximal static exercise. Improvements were made to the system-based monitoring paradigm to facilitate online fatigue assessment and more accurately represent changes occurring in the NMS. This work presents the first step toward evaluating the clinical viability of a system-based monitoring strategy for assessing fatigue by comparing its performance with traditional fatigue measures. Ultimately, the ability to monitor and assess fatigue has important implications for preventing neuromuscular injury, optimizing training loads, and guiding effective, individualized treatment strategies for rehabilitation.

## Data Availability

The data presented in this study are available on request from the corresponding author. The data are not publicly available due to continuing study by the authors.

## Abbreviations

The following abbreviations are used in this manuscript:

sEMG	Surface Electromyography
MVC	Maximum Voluntary Contraction
RPE	Rating of Perceived Exertion
FSI	Freshness Similarity Index
RMS	Root Mean Square
TFD	Time Frequency Distribution
ARMAX	Autoregressive Moving Average Model with Exogenous Inputs
RM-ANOVA	Repeated Measures Analysis of Variance
rmcorr	Repeated Measures Correlation
MU	Motor Unit
NMS	Neuromusculoskeletal System
